# Characterizing the protective effects of SHLP2, a mitochondrial-derived peptide, in macular degeneration

**DOI:** 10.1038/s41598-018-33290-5

**Published:** 2018-10-11

**Authors:** Sonali Nashine, Pinchas Cohen, Anthony B. Nesburn, Baruch D. Kuppermann, M. Cristina Kenney

**Affiliations:** 1 Department of Ophthalmology, Gavin Herbert Eye Institute, University of California Irvine, Irvine, CA USA; 2 Leonard Davis School of Gerontology, University of Southern California, Los Angeles, CA USA; 3 Cedars-Sinai Medical Center, Los Angeles, CA USA; 4 Department of Pathology and Laboratory Medicine, University of California Irvine, Irvine, CA USA

## Abstract

Mitochondrial-derived peptides (MDPs) are rapidly emerging therapeutic targets to combat development of neurodegenerative diseases. SHLP2 (small humanin-like peptide 2) is a newly discovered MDP that is coded from the *MT-RNR2* (Mitochondrially encoded 16S rRNA) gene in mitochondrial DNA (mtDNA). In the current study, we examined the biological consequences of treatment with exogenously-added SHLP2 in an *in vitro* human transmitochondrial age-related macular degeneration (AMD) ARPE-19 cell model. In AMD cells, we observed significant down-regulation of the MDP-coding *MT-RNR2* gene, and remarkably reduced levels of all five oxidative phosphorylation (OXPHOS) complex I-V protein subunits that are involved in the electron transport chain; these results suggested mitochondrial toxicity and abnormal OXPHOS complex protein subunits’ levels in AMD cells. However, treatment of AMD cells with SHLP2: (1) restored the normal levels of OXPHOS complex protein subunits, (2) prevented loss of viable cells and mitochondria, (3) increased the number of mtDNA copies, (4) induced anti-apoptotic effects, and (5) attenuated amyloid-β-induced cellular and mitochondrial toxicity. Cumulatively, our findings established the protective role of SHLP2 in AMD cells *in vitro*. In conclusion, this novel study supports the merit of SHLP2 in the treatment of AMD, a primary retinal disease that is a leading cause of blindness among the elderly population in the United States as well as worldwide.

## Introduction

Loss of retinal pigment epithelium (RPE) cells is a hallmark of macular degeneration, a devastating retinal degenerative disease that can manifest phenotypically as geographic atrophy (dry age-related macular degeneration (AMD)) or choroidal neovascularization (exudative/wet AMD)^1^. RPE cell death is a critical point for development and testing of potential therapeutic interventions.

It is evident from our previous studies that the effects of mitochondrial dysfunction in macular degeneration far exceed perturbed cellular respiration^2^. Our recent studies have shown that mitochondria from AMD patients are unhealthy and AMD mitochondrial DNA (mtDNA) when placed in Rho0 (mtDNA-deficient) ARPE-19 cells, contribute to a wide variety of cell damaging events^3^. In the transmitochondrial ARPE-19 cybrid model used in this study, all cells possess identical nuclei, derived from ARPE-19 cell lines, and mtDNA derived either from AMD patients (AMD cybrids) or age-matched normal subjects (normal cybrids). Therefore, this model helps evaluate differences between mitochondria from diseased versus normal subjects. In addition to influencing cellular respiration and energy turnover, AMD mitochondria also affect the expression of nuclear-encoded genes that have been linked to AMD pathology, such as those involved in mitochondrial replication/transcription, apoptosis, autophagy, inflammation, angiogenesis, and complement pathways^4^.

Mitochondrial-derived peptides (MDPs) that are coded from distinct open-reading frames (ORFs) within the mtDNA are known to provide a wide array of protective effects, including neuroprotective, cytoprotective, anti-oxidant, anti-inflammatory, and metabolic properties^5–7^. Of the several known MDPs, Humanin which is coded from the *MT-RNR2* (Mitochondrially encoded 16S rRNA) gene was the first-discovered MDP and has been very well-characterized as a cytoprotective peptide in a plethora of disease models including neurodegenerative and retinal diseases^5^. Furthermore, our recent work highlighted the role of Humanin-G, a single amino acid Humanin variant, in protecting AMD transmitochondrial RPE cybrids against cellular and mitochondrial damage^4^. In addition to Humanin, the 16S region of the mtDNA also codes for six additional MDPs referred to as small humanin-like peptides (SHLP1, SHLP2, SHLP3, SHLP4, SHLP5, and SHLP6) that are 24–38 amino acids in length. Of these, SHLP2 is a 26 amino acid peptide (Molecular Weight-3017.54 D) that has been demonstrated to modulate cellular and mitochondrial functions in a recent study^8^. In that study, SHLP2 increased the number of viable cells and improved mitochondrial bioenergetics in pancreatic cell lines. Another study demonstrated that SHLP2 mediates chaperone-like effects^9^.

In the current study, we hypothesized that SHLP2 will preserve AMD cells and mitochondria from toxicity. Herein, we characterized the ability of SHLP2 to protect retinal cells in AMD, using the transmitochondrial ARPE-19 cybrid cells that contain identical nuclei (derived from mtDNA-deficient (Rho0) ARPE-19 cells) but differ in mtDNA, which is derived either from AMD patients (AMD cybrids) or normal subjects (normal cybrids). Therefore, each cybrid cell line represented one individual patient. Our study revealed that administration of SHLP2 confers cellular and mitochondrial protection in AMD, indicating the therapeutic potential of SHLP2 for treatment of AMD.

## Results

### *MT-RNR2* gene is down-regulated in AMD cybrids

**Figure 1:**
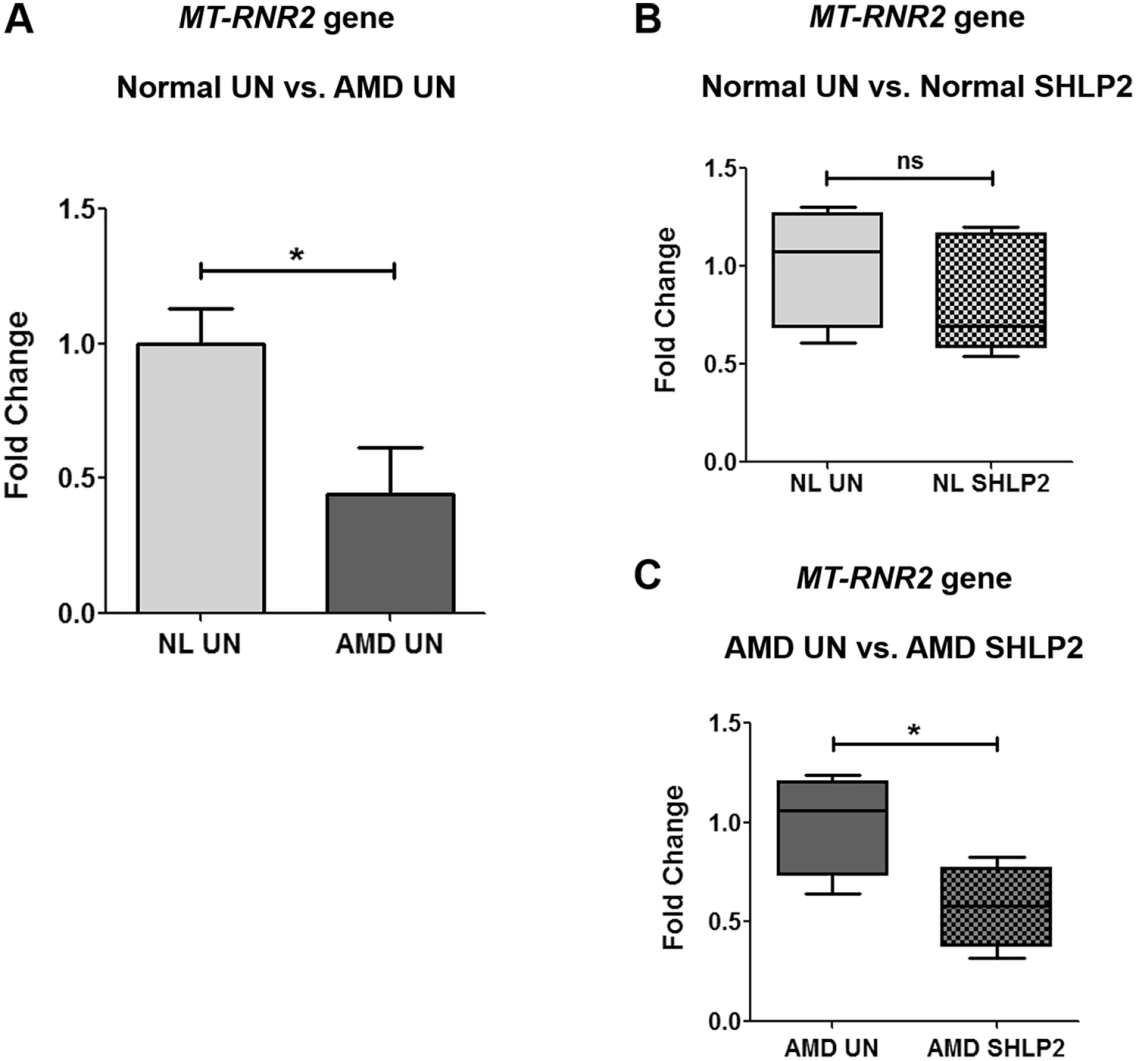
*MT-RNR2* gene is down-regulated in AMD. (**A**) AMD cybrids had reduced *MT-RNR2* gene expression (56% decrease, P = 0.03, n = 5) compared to age-matched normal (NL) cybrids. Administration of SHLP2 decreased *MT-RNR2* gene expression in: (**B**) normal (16% decrease, P = 0.43, n = 5) and (**C**) AMD (42.3% decrease, P = 0.04, n = 4) cybrids. Data are presented as Mean ± SEM and normalized to untreated (UN) normal cybrids which were assigned a value of 1. Experiments were performed at the 72 hr time-point.

To examine the expression of *MT-RNR2* gene that contains small open-reading frames (ORFs) for MDPs such as Humanin and SHLPs, we performed quantitative real-time polymerase chain reaction (qRT-PCR) using *MT-RNR2* targeting TaqMan gene expression assays. We observed a 56% decrease in *MT-RNR2* gene expression in untreated AMD cybrids (*0*.*44* ± *0*.*17 a*.*u*. (*arbitrary unit*)) compared to untreated normal cybrids (*1* ± *0*.*13 a*.*u*.) (*P* = *0*.*03*, *n* = *5*) (Fig. 1A). This suggests that down-regulation of *MT-RNR2* gene might contribute to reduced production of MDPs which in turn leads to reduced cytoprotective effects and eventually unhealthy AMD cybrid cells. Furthermore, treatment with SHLP2 caused no significant change in the expression of *MT-RNR2* gene in normal cybrids (*NL UN: 1* ± *0*.*13 a*.*u*., *NL SHLP2: 0*.*84* ± *0*.*14 a*.*u*.; *P* = *0*.*43*, *n* = *5*) (Fig. 1B). In contrast, AMD cybrids showed *42.3%* decline in *MT-RNR2* expression after treatment with SHLP2 (*AMD UN: 1* ± *0*.*128 a*.*u*., *AMD SHLP2: 0*.*577* ± *0*.*10 a*.*u*.; *P* = *0*.*04*, *n* = *4*) (Fig. 1C). This response to SHLP2 may indicate the presence of a feedback loop that counterbalances the production of MDPs within the cellular system. This counterbalancing effect was specific to AMD cybrids and was not observed in normal cybrids.

### SHLP2 stabilizes the protein levels of mitochondrial OXPHOS complex subunits

**Figure 2:**
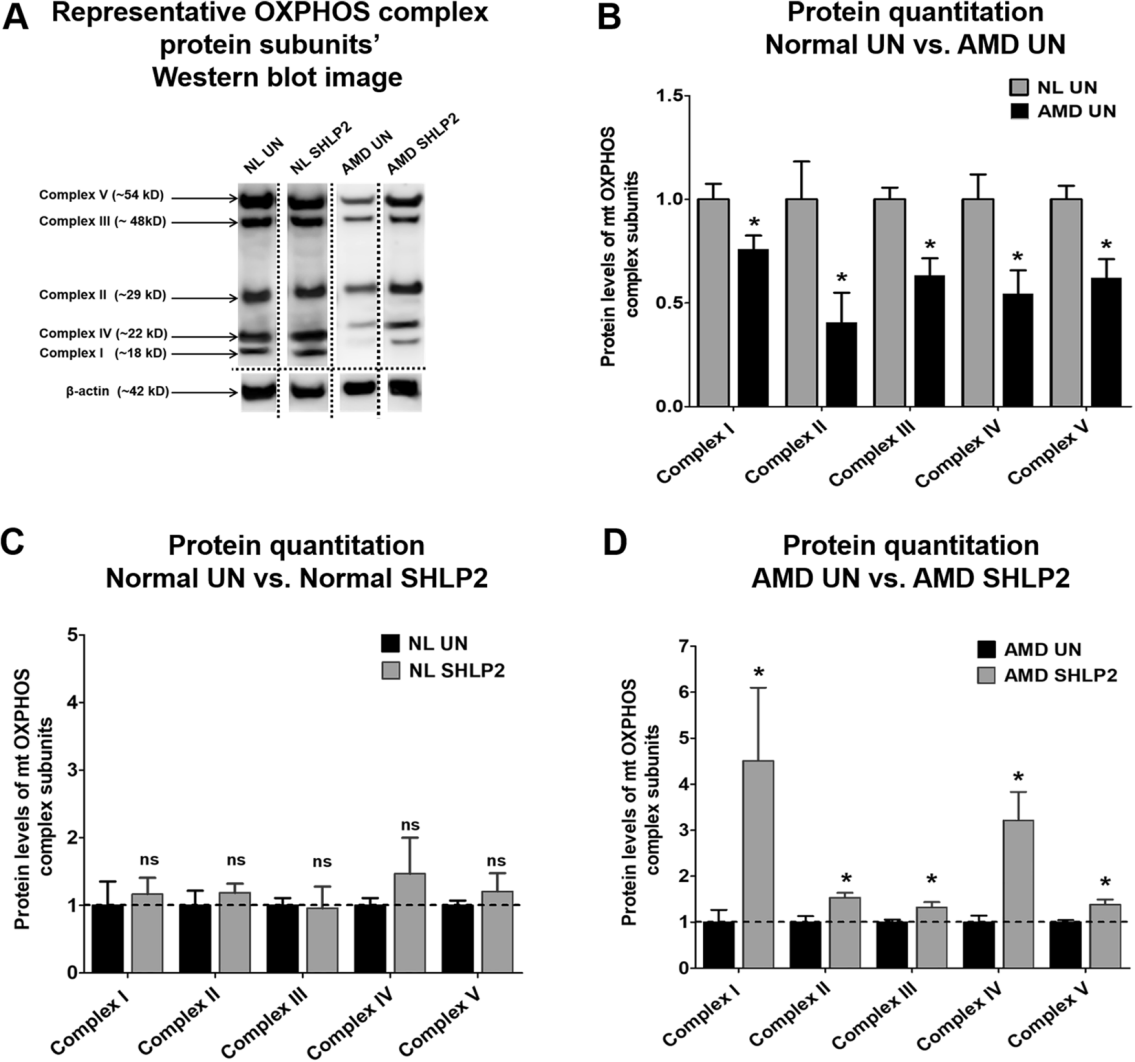
SHLP2 restores the OXPHOS complex I-V subunit protein levels to normal. (**A**) This figure shows representative Western blot images for OXPHOS subunit proteins and the loading control β-actin. The dotted lines demarcate the borders of cropped images for each of the four groups i.e., Normal untreated (NL UN), Normal SHLP2-treated, AMD untreated (AMD UN), and AMD SHLP2-treated. Full-length blots are presented in Supplementary Figures S1A, S1B, S2A and S2B. Loading control β-actin was run on the same gel. (**B**) A drastic decline in the protein levels of OXPHOS Complex I subunit (24%, P = 0.04, n = 5), Complex II subunit (59%, P = 0.04, n = 4), Complex III subunit (37%, P = 0.013, n = 3–4), Complex IV subunit (46%, P = 0.04, n = 3–4), and Complex V subunit (38%, P = 0.01, n = 4) was observed in AMD cybrids compared to those in normal cybrid cells. (**C**) The levels of OXPHOS complex proteins remained unchanged in normal cybrids treated with SHLP2 (P > 0.05, n = 4–5), compared to their untreated counterparts. (**D**) Addition of SHLP2 to AMD cybrids increased protein levels of Complex I subunit (350.8%, P = 0.028, n = 3–5), Complex II subunit (54%, P = 0.01, n = 5), Complex III subunit (32%, P = 0.03, n = 5), Complex IV subunit (221%, P = 0.03, n = 3–4), Complex V subunit (38%, P = 0.03, n = 3–4). Data are presented as Mean ± SEM. Data in Fig. B and Fig. C are normalized to untreated normal cybrids which were assigned a value of 1. Data in Fig. D are normalized to untreated AMD cybrids which were assigned a value of 1. Experiments were performed at the 72 hr time-point.

Since a prominent function of mitochondria is ATP production via the oxidative phosphorylation (OXPHOS) pathway, we next investigated protein levels of OXPHOS complex subunits. Western blotting analyses comparing protein levels of Complex I subunit (NADH-coenzyme Q oxidoreductase), Complex II subunit (Succinate-coenzyme Q oxidoreductase), Complex III subunit (Coenzyme Q-cytochrome c oxidoreductase), Complex IV subunit (Cytochrome c oxidase), and Complex V subunit (ATP synthase) were performed for untreated and SHLP2-treated normal and AMD cybrids (Fig. 2A) (Fig. S1A, S1B, S2A, and S2B). As shown in Fig. 2B, AMD cybrids had significant decreases in the protein levels of OXPHOS Complex I subunit (*24% lower*; *NL UN: 1* ± *0*.*075 a*.*u*.; *AMD UN: 0*.*76* ± *0*.*067 a*.*u*.; *P* = *0*.*04*, *n* = *5*), Complex II subunit (*59% lower*; *NL UN: 1* ± *0*.*18 a*.*u*.; *AMD UN: 0*.*41* ± *0*.*14 a*.*u*.; *P* = *0*.*04*, *n* = *4*), Complex III subunit (*37% lower*; *NL UN: 1* ± *0*.*057 a*.*u*.; *AMD UN: 0*.*63* ± *0*.*08 a*.*u*.; *P* = *0*.*013*, *n* = *3–4*), Complex IV subunit (*46% lower*; *NL UN: 1* ± *0*.*12 a*.*u*.; *AMD UN: 0*.*54* ± *0*.*11 a*.*u*.; *P* = *0*.*04*, *n* = *3–4*), and Complex V subunit (*38% lower*; *NL UN: 1* ± *0*.*06 a*.*u*.; *AMD UN: 0*.*62* ± *0*.*089 a*.*u*.; *P* = *0*.*01*, *n* = *4*) compared to normal cybrids.

Addition of SHLP2 to normal cybrids did not result in any significant change in the protein levels of OXPHOS complex protein subunits (Fig. 2C): Complex I subunit (*NL UN: 1* ± *0*.*35 a*.*u*. *vs*. *NL SHLP2-treated: 1*.*17* ± *0*.*24 a*.*u*.; *P* = *0*.*69*, *n* = *4–5*), Complex II subunit (*NL UN: 1* ± *0*.*22 a*.*u*. *vs*. *NL SHLP2-treated: 1*.*19* ± *0*.*13 a*.*u*.; *P* = *0*.*46*, *n* = *4–5*), Complex III subunit (*NL UN: 1* ± *0*.*11 a*.*u*. *vs*. *NL SHLP2-treated: 0*.*96* ± *0*.*32 a*.*u*.; *P* = *0*.*91*, *n* = *4–5*), Complex IV subunit (*NL UN: 1* ± *0*.*11 a*.*u*. *vs*. *NL SHLP2-treated: 1*.*47* ± *0*.*53 a*.*u*.; *P* = *0*.*47*, *n* = *4–5*), and Complex V subunit (*NL UN: 1* ± *0*.*07 a*.*u*. *vs*. *NL SHLP2-treated: 1*.*21* ± *0*.*27 a*.*u*.; *P* = *0*.*48*, *n* = *4*).

However, in AMD cybrids, SHLP2 treatment led to a drastic increase in the levels of all five OXPHOS complex protein subunits (Fig. 2D): Complex I subunit (350.8% increase; *AMD UN: 1* ± *0*.*268 a*.*u*. *vs*. *AMD SHLP2-treated: 4*.*508* ± *1*.*59 a*.*u*.; *P* = *0*.*028*, *n* = *3–5*), Complex II subunit (54% increase; *AMD UN: 1* ± *0*.*13 a*.*u*. *vs*. *AMD SHLP2-treated: 1*.*54* ± *0*.*10 a*.*u*.; *P* = *0*.*01*, *n* = *5*), Complex III subunit (32% increase; *AMD UN: 1* ± *0*.*05 a*.*u*. *vs*. *AMD SHLP2-treated: 1*.*32* ± *0*.*11 a*.*u*.; *P* = *0*.*03*, *n* = *5*), Complex IV subunit (221% increase; *AMD UN: 1* ± *0*.*14 a*.*u*. *vs*. *AMD SHLP2-treated: 3*.*21* ± *0*.*62 a*.*u*.; *P* = *0*.*03*, *n* = *3–4*), and Complex V subunit (38% increase; *AMD UN: 1* ± *0*.*04 a*.*u*. *vs*. *AMD SHLP2-treated: 1*.*38* ± *0*.*10 a*.*u*.; *P* = *0*.*03*, *n* = *3–4*).

These results suggest that the compromised OXPHOS complex protein subunits’ levels observed in untreated AMD cybrids can be restored through treatment with SHLP2.

### SHLP2 prevents loss of mitochondria

**Figure 3:**
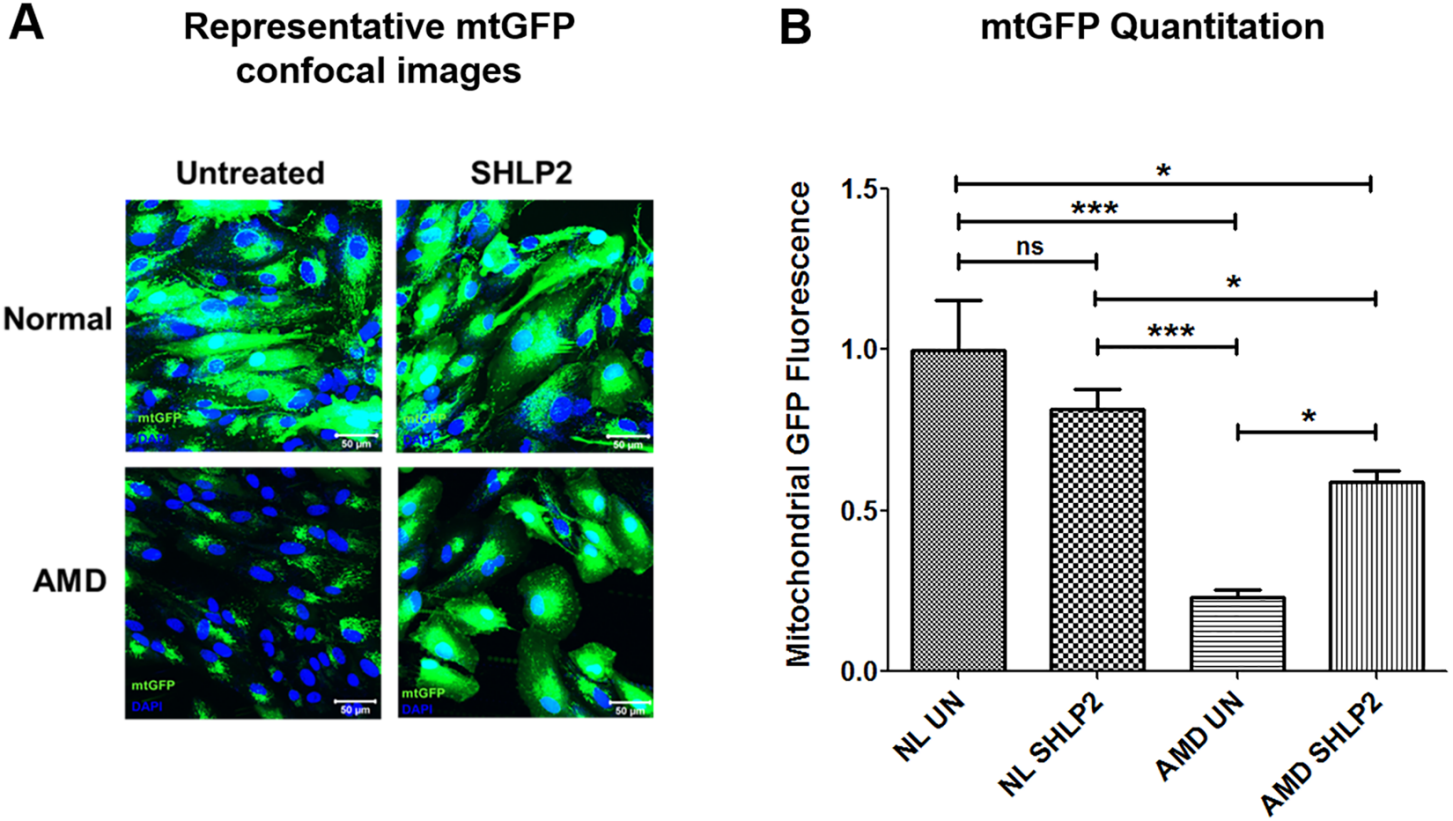
SHLP2 prevents loss of mitochondria. (**A**) Representative confocal images showing: (1) Relatively abundant mtGFP staining in normal (NL) untreated (UN) (top left panel) and NL SHLP2-treated (top right panel) cybrid cells, and (2) drastic increase in mtGFP staining in SHLP2-treated AMD cybrids (lower right panel) compared to AMD UN cybrids (lower left panel). Scale bar equals 50 µm; Green color represents mitochondrial GFP (mtGFP); Blue color represents DAPI (nuclear stain)). (**B**) Quantitation of the images showed that SHLP2 increased mtGFP fluorescence by 153.02% (P < 0.05, n = 4–5) in the cytoplasm of AMD cybrid cells. All mtGFP fluorescence intensities within a group were normalized to DAPI. Data are presented as Mean ± SEM and normalized to untreated normal cybrids which were assigned a value of 1. Experiments were performed at the 72 hr time-point.

To examine the effects of SHLP2 on mitochondrial abundance in AMD, cells were stained with a fluorescent probe that specifically targets mitochondria, followed by confocal imaging (Fig. 3A). SHLP2 led to a 153.02% increase in mtGFP fluorescence intensity in AMD cybrids (*AMD UN: 0*.*232* ± *0*.*026 a*.*u*.; *AMD SHLP2: 0*.*587* ± *0*.*038 a*.*u*.; *P* < *0*.*05*, *n* = *4–5*) (Fig. 3B–bar 3 vs. bar 4). No significant difference in fluorescence signal was observed between untreated and SHLP2-treated normal cybrids (*NL UN: 1* ± *0*.*15 a*.*u*.; *NL SHLP2: 0*.*816* ± *0*.*059 a*.*u*.; *P* > *0*.*05*, *n* = *4–5*) (Fig. 3B–bar 1 vs. bar 2). These results indicate that SHLP2 enhances mitochondrial staining in AMD cybrid cells.

### SHLP2 increases mtDNA copy number and *PGC-1α* gene expression

**Figure 4:**
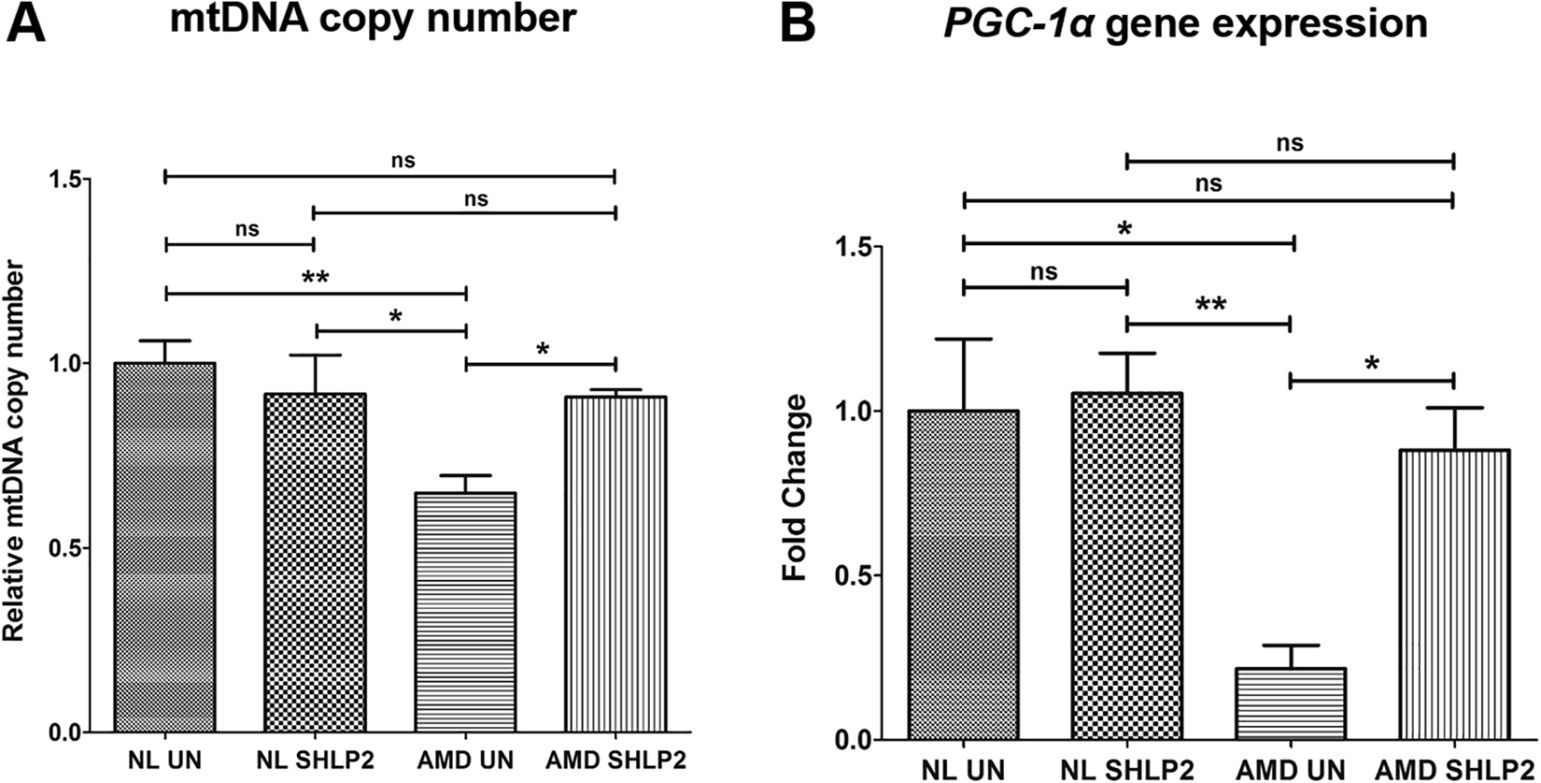
SHLP2 increases mtDNA copy number and *PGC-1α* gene expression. (**A**) Bar graph showing the effects of SHLP2 treatment on mtDNA copy number in normal (NL) and AMD cybrids. No significant difference in mtDNA copy number was observed between untreated (UN) NL and SHLP2-treated NL cybrids (bar 1 vs. bar 2). Significant increase in mtDNA copy number was observed in SHLP2-treated AMD cybrids compared to untreated AMD cybrids (40.3%, P < 0.05, n = 4) (bar 3 vs. bar 4). (**B**) Bar graph showing the effects of SHLP2 treatment on *PGC-1α* gene expression in normal (NL) and AMD cybrids. No significant difference in *PGC-1α* gene expression was observed between untreated (UN) NL and SHLP2-treated NL cybrids (bar 1 vs. bar 2). However, treatment with SHLP2 caused significant increase in *PGC-1α* gene expression in AMD cybrids compared to their untreated counterparts (307.87%, P < 0.05, n = 4) (bar 3 vs. bar 4). Data are presented as Mean ± SEM and normalized to untreated normal cybrids which were assigned a value of 1. Experiments were performed at the 72 hr time-point.

Next, the effects of SHLP2 on mtDNA copy number and *PGC-1α* gene expression levels were examined using qRT-PCR. SHLP2 substantially increased mtDNA copy number in AMD cybrids compared to their untreated counterparts (40.3% increase; *AMD UN: 0*.*648* ± *0*.*047 a*.*u*. *vs*. *AMD SHLP2-treated: 0*.*909* ± *0*.*019 a*.*u*.; *P* < *0*.*05*, *n* = *4*. No significant difference in mtDNA copy number was observed between untreated and SHLP2-treated normal cybrids (*NL UN: 1* ± *0*.*06 a*.*u*.; *NL SHLP2: 0*.*916* ± *0*.*105 a*.*u*.; *P* > *0*.*05*, *n* = *3*) (Fig. 4A).

Furthermore, SHLP2-treated AMD cybrids showed up-regulated *PGC-1α* gene expression in comparison to untreated AMD cybrids (307.87% increase; *AMD UN: 0*.*216* ± *0*.*071 a*.*u*. *vs*. *AMD SHLP2-treated: 0*.*881* ± *0*.*129 a*.*u*.; *P* < *0*.*05*, *n* = *4*. No significant difference in *PGC-1α* expression was observed between untreated and SHLP2-treated normal cybrids (*NL UN: 1* ± *0*.*219 a*.*u*.; *NL SHLP2: 1*.*053* ± *0*.*122 a*.*u*.; *P* > *0*.*05*, *n* = *4*) (Fig. 4B).

In summary, these results indicate that SHLP2 increases mtDNA copy number and up-regulates *PGC-1α* gene in AMD cybrid cells.

### SHLP2 prevents loss of viable cells and reduces apoptosis

**Figure 5:**
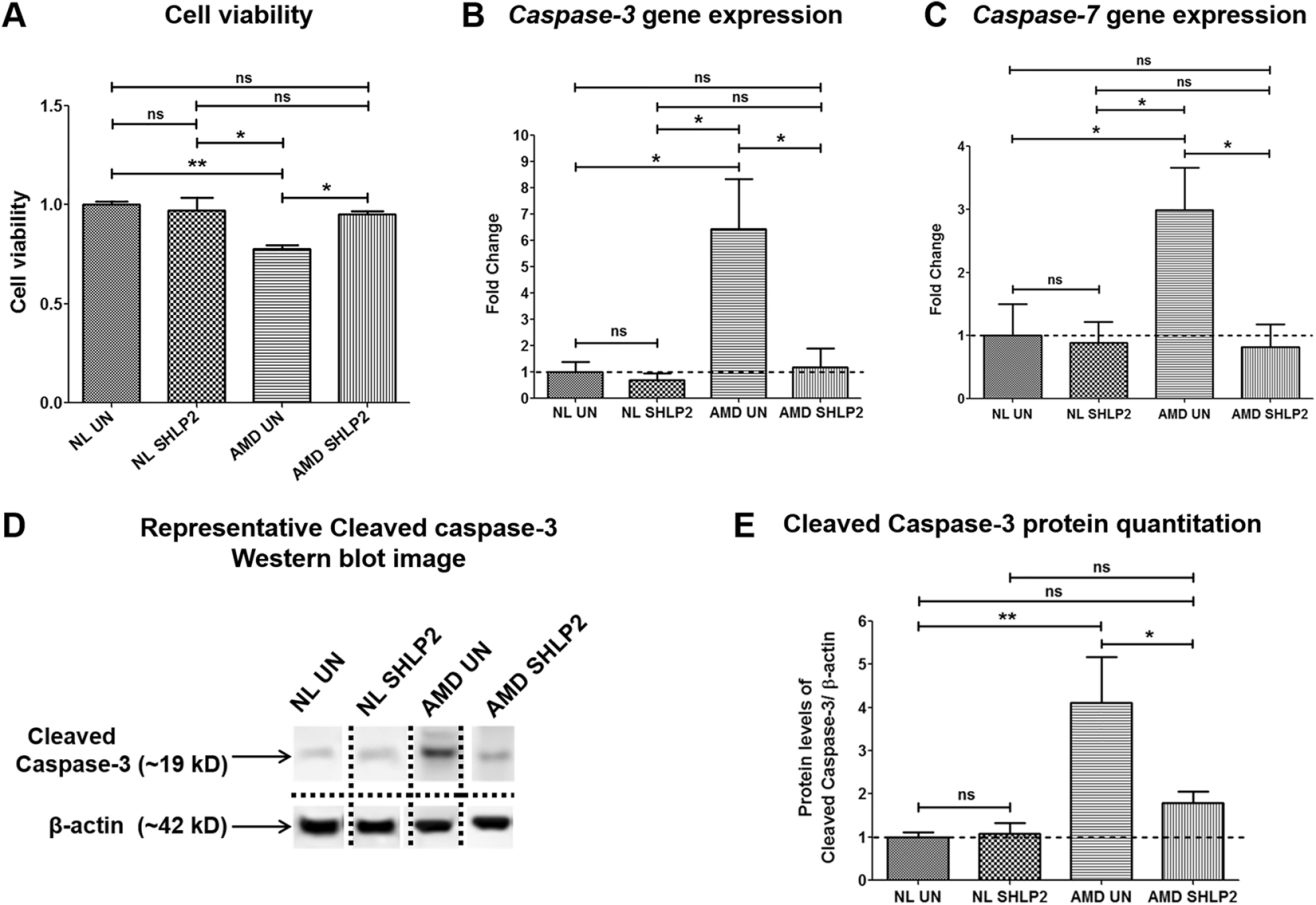
SHLP2 prevents loss of viable cells and reduces apoptosis. (**A**) Treatment with SHLP2 did not bring about any differences in cell viability between the untreated normal (NL UN) and SHLP2-treated normal (NL SHLP2) cybrids (P > 0.05, n = 4). However, SHLP2 protected AMD cybrids from loss of viable cells (21.79% increase, P < 0.05, n = 4). (**B**) SHLP2 treatment significantly decreased the expression of *Caspase-3* gene (81.8%, P < 0.05, n = 4) in AMD cybrids and caused no change in normal cybrids. (**C**) SHLP2 down-regulated *Caspase-7* gene (72.48%, P < 0.05, n = 4–5) in AMD cybrids with no effect in normal cybrids. (**D**) Representative Western blot images for cleaved Caspase-3 protein and β-actin. The dotted lines demarcate the borders of cropped images for each of the four groups i.e., Normal untreated (NL UN), Normal SHLP2-treated, AMD untreated (AMD UN), and AMD SHLP2-treated. Full-length blots are presented in Supplementary Figs S3A, S3B, S4A, and S4B. Loading control β-actin was run on the same gel. (**E**) Quantitation graphs showed that SHLP2 reduced Cleaved Caspase-3 protein levels in AMD cybrids by 56.45% (P < 0.05, n = 4–5). No significant difference was observed between untreated and SHLP2-treated normal cybrids (P > 0.05, n = 4–5). Data are presented as Mean ± SEM and normalized to normal cybrids which were assigned a value of 1. Experiments were performed at the 72 hr time-point.

 Differences in cell viability between untreated and SHLP2-treated AMD and normal groups were examined using MTT assay. Treatment with SHLP2 prevented loss of viable cells and led to a 21.79% increase in viable cell numbers in AMD cybrids (*AMD UN: 0*.*78* ± *0*.*019 a*.*u*.; *AMD SHLP2: 0*.*95* ± *0*.*014 a*.*u*.; *P* < *0*.*05*, *n* = *4*) (Fig. 5A–bar 3 vs. bar 4). No significant difference in cell viability was found between untreated and SHLP2-treated normal groups (*NL UN: 1* ± *0*.*0156 a*.*u*.; *NL SHLP2: 0*.*97* ± *0*.*06 a*.*u*.; *P* > *0*.*05*, *n* = *4*) (Fig. 5A–bar 1 vs. bar 2).

The mechanism by which SHLP2 enhances cell viability was examined by measuring gene expression levels of effector caspases (−3 and −7) which are universal apoptotic cell markers. In AMD cybrids, SHLP2 significantly down-regulated the *Caspase-3* gene by 81.8% (*AMD UN: 6*.*43* ± *1*.*90 a*.*u*.; *AMD SHLP2: 1*.*17* ± *0*.*73 a*.*u*.; *P* < *0*.*05*, *n* = *4*) (Fig. 5B–bar 3 vs. bar 4), and *Caspase-7* gene by 72.48% (*AMD UN: 2*.*98* ± *0*.*67 a*.*u*.; *AMD SHLP2: 0*.*82* ± *0*.*36 a*.*u*.; *P* < *0*.*05*, *n* = *4–5*) (Fig. 5C–bar 3 vs. bar 4). No appreciable differences in gene expression were observed between untreated and SHLP2-treated normal cybrids for either *Caspase-3* (*NL UN: 1* ± *0*.*398 a*.*u*.; *NL SHLP2: 0*.*69* ± *0*.*26 a*.*u*.; *P* > *0*.*05*, *n* = *4–5*) (Fig. 5B–bar 1 vs. bar 2) or *Caspase-7* (*NL UN: 1* ± *0*.*49 a*.*u*.; *NL SHLP2: 0*.*89* ± *0*.*33 a*.*u*.; *P* > *0*.*05*, *n* = *4–5*) (Fig. 5C–bar 1 vs. bar 2).

To further investigate the effects of SHLP2 on Caspase-3 protein activation, cleaved Caspase-3 protein levels were detected using immunoblotting (Fig. 5D) (Fig. S3A, S3B, S4A, and S4B). In AMD cybrids, SHLP2 decreased the cleaved Caspase-3 protein levels by 56.45% (*AMD UN: 4*.*11* ± *1*.*07 a*.*u*.; *AMD SHLP2: 1*.*79* ± *0*.*26 a*.*u*.; *P* < *0*.*05*, *n* = *4–5*) (Fig. 5D–bar 3 vs. bar 4). No significant difference in cleaved Caspase-3 protein levels was observed between untreated and SHLP2-treated normal cybrids (*NL UN: 1* ± *0*.*11 a*.*u*.; *NL SHLP2: 1*.*08* ± *0*.*24 a*.*u*.; *P* > *0*.*05*, *n* = *4-5*) (Fig. 5E–bar 1 vs. bar 2).

### SHLP2 protects against Amyloid-β-induced cell death

**Figure 6:**
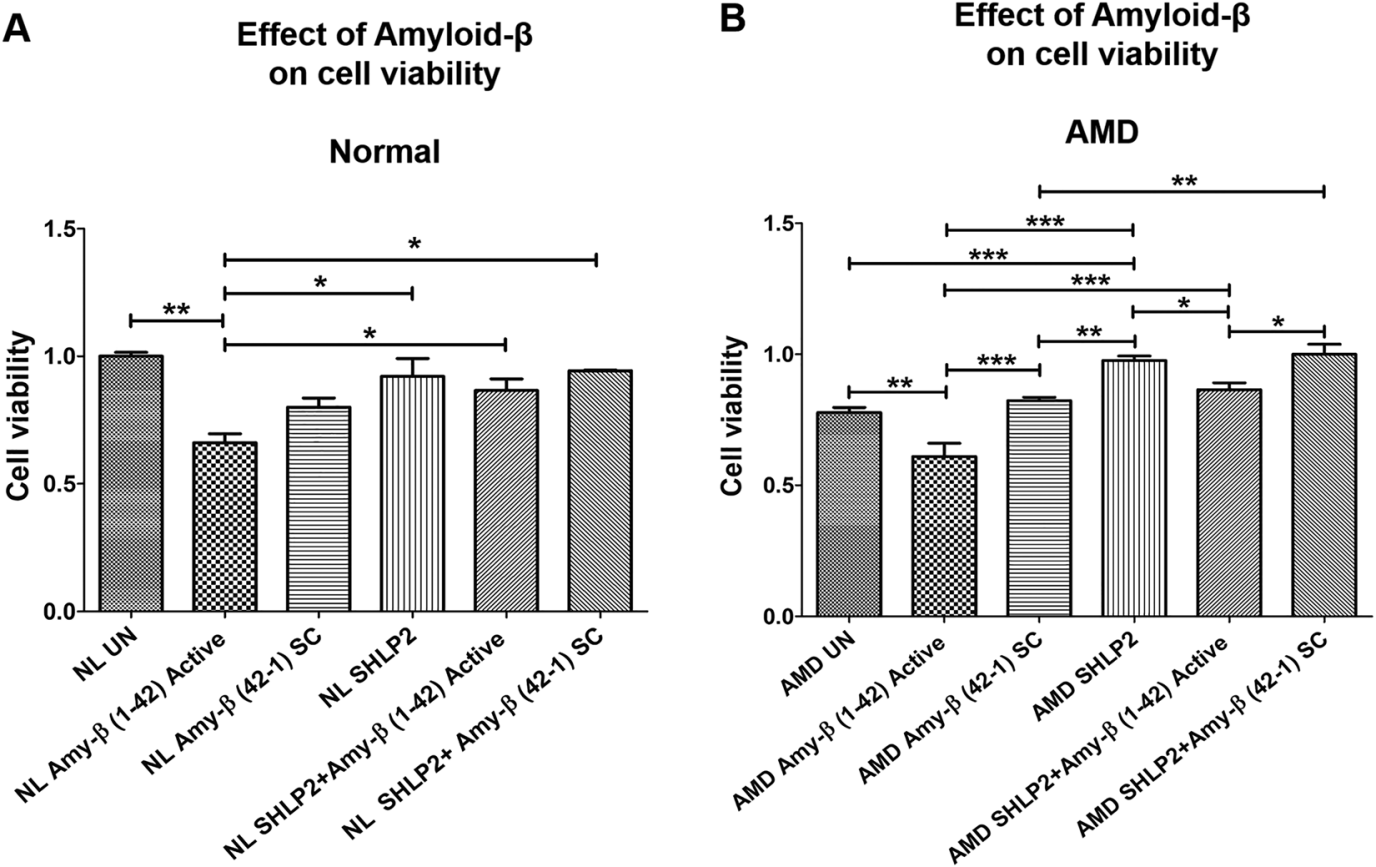
SHLP2 protects against Amyloid-β-induced cell death. (**A**) Addition of amyloid-β_1–42_ active peptide diminished the number of viable cells in normal (NL) cybrid cells compared to their untreated (UN) counterparts (bar 1 vs. bar 2). No significant difference in cell viability was observed between untreated and amyloid-β_42–1_ SC-treated NL cybrids (bar 1 vs. bar 3). Treatment with SHLP2 significantly increased the cell viability in amyloid-β_1–42_-treated NL (bar 5) and amyloid-β_42–1_ SC-treated NL cybrids (bar 6) compared to the ones treated with amyloid-β_1–42_ alone (bar 2). (**B**) Amyloid-β_1–42_-treated AMD cybrids showed reduced number of viable cells compared to the untreated group (bar 1 vs. bar 2) and the amyloid-β_42–1_ SC-treated group (bar 2 vs. bar 3). SHLP2 pretreatment resulted in a drastic increase in cell viability in amyloid-β_1–42_-treated (bar 5) and amyloid-β_42–1_ SC-treated (bar 6) AMD cybrids compared to the AMD cybrids treated with amyloid-β_1–42_ alone (bar 2). Data are presented as Mean ± SEM and normalized to untreated-normal cybrids which were assigned value of 1. Experiments were performed at the 72 hr time-point.

The role of SHLP2 against the deleterious effects mediated by amyloid-β insult was characterized by exposing cybrid cells to amyloid-β_1–42_ (active form) and amyloid-β_42-1_ (inactive scrambled control (SC)) peptides and then measuring numbers of viable cells. As expected and as previously established, addition of amyloid-β_1–42_ dramatically decreased cell viability in both normal (*NL UN: 1* ± *0*.*016 a*.*u*. *vs*. *NL amyloid-β*_*1*–*42*_*: 0*.*66* ± *0*.*035* *a*.*u*. *P* < *0*.*01*, *n* = *3–4*) (Fig. 6A–bar 1 vs. bar 2) and AMD cybrids (*AMD UN: 0*.*777* ± *0*.*019 a*.*u*. *vs*. *AMD amyloid-β*_*1–42*_*: 0*.*61* ± *0*.*051 a*.*u*.; *P* < *0*.*01*, *n* = *3–4*) (Fig. 6B–bar 1 vs. bar 2). We speculate that a slight drop in cell viability in NL Amy-β SC (Fig. 6A bar 1 vs. bar 3) was due to addition of scrambled control. However, the difference was not significant i.e., P-value = 0.16. Similar drop in cell viability between NL UN and NL Amy-β SC was observed in our recent manuscript which demonstrated the effect of Humanin G on AMD cybrids. Furthermore, pretreatment with SHLP2 prior to addition of amyloid β_1–42_ (active form) led to a significant increase in the number of viable cells in both normal (*30*.*30%*; *NL amyloid-β*_*1*–*42*_*: 0*.*66* ± *0*.*035 a*.*u*. *vs*. *NL SHLP2* + *amyloid-β*_*1*–*42*_*: 0*.*86* ± *0*.*046 a*.*u*.; *P* < *0*.*05*, *n* = *3*) (Fig. 6A–bar 2 vs. bar 5), and AMD cybrids (*40*.*98%*; *AMD amyloid-β*_*1*–*42*_*: 0*.*61* ± *0*.*051 a*.*u*. *vs*. *AMD SHLP2* + *amyloid-β*_*1*–*42*_*: 0*.*86* ± *0*.*026 a*.*u*.; *P* < *0*.*001*, *n* = *3-4*) (Fig. 6B–bar 2 vs. bar 5). No significant difference was observed between NL amyloid-β_42–1_ SC (*0*.*80* ± *0*.*036 a*.*u*.) and NL SHLP2 + amyloid-β_42–1_ SC (*0*.*94* ± *0*.*003 a*.*u*.) (*P* > *0*.*05*, *n* = *3*) (Fig. 6A–bar 3 vs. bar 6). However, pretreatment with SHLP2 increased cell viability by 21.95% in amyloid-β_42–1_ SC-treated AMD cybrids (*AMD amyloid-β*_*42*–*1*_ *SC: 0*.*82* ± *0*.*013 a*.*u*. *vs*. *AMD SHLP2* + *amyloid-β*_*42*–*1*_ *SC: 1*.*00* ± *0*.*038 a*.*u*.; *P* < *0*.*01*, *n* = *4*) (Fig. 6B–bar 3 vs. bar 6). This suggests that SHLP2 can rescue cybrids from amyloid-β-induced cell loss.

### SHLP2 protects against Amyloid-β-induced mitochondrial damage

**Figure 7:**
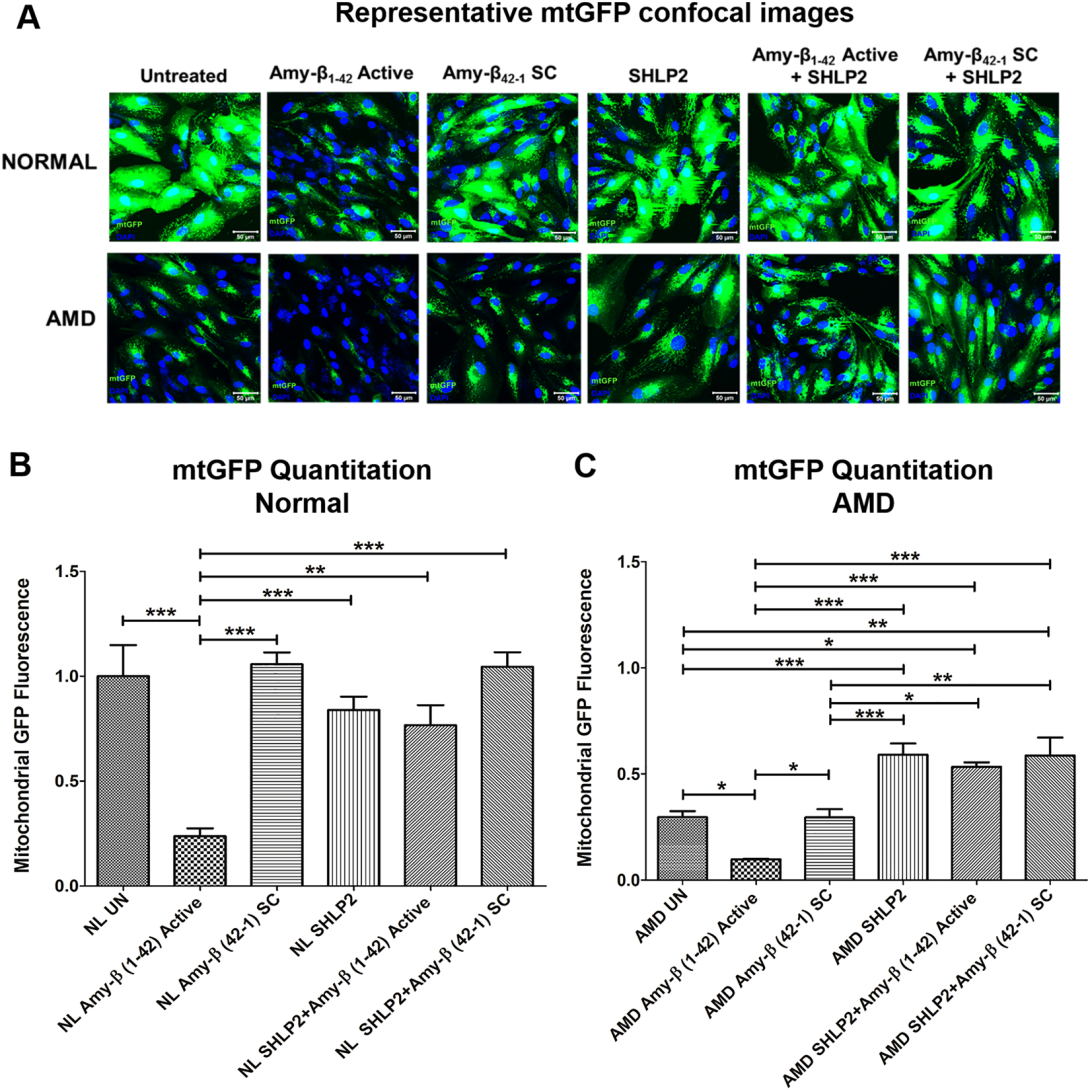
SHLP2 protects against Amyloid-β-induced mitochondrial damage. (**A**) Representative confocal images showing mtGFP staining in untreated, amyloid-β_1–42_-treated, amyloid-β_42-1_ SC-treated, SHLP2-treated, SHLP2 + amyloid-β_1–42_-treated, and SHLP2 + amyloid-β_42-1_-treated normal and AMD cybrid cells. (**B**) Quantitation graphs for normal cybrids, (**C**) Quantitation graphs for AMD cybrids. Scale bar equals 50 µm; Green color represents mitochondrial GFP (mtGFP); Blue color represents DAPI (nuclear stain)). This experiment showed: (1) reduced mtGFP signal in amyloid-β_1–42_-treated (amyloid-β_1–42_ Active) normal (7A top panel-2^nd^ image, (77% decrease- 7B)) and AMD (7A bottom panel–2^nd^ image; 65% decrease-7C) cybrids compared to their untreated counterparts (7A-NL UN: top panel-1^st^ image; AMD UN: bottom panel-1^st^ image); (2) No significant difference in mtGFP signal was observed between untreated and amyloid-β_42–1_ SC-treated normal (7A-top panel-3^rd^ image) or AMD (7A-bottom panel-3^rd^ image) cybrids; (3) Addition of SHLP2 increased mtGFP signal in amyloid-β_1–42_-treated (amyloid-β_1–42_ Active) normal (7A top panel-5^th^ image; 223.2% increase-7B) but caused no significant change in amyloid-β_42–1_ SC-treated normal (7A top panel–6^th^ image); (4) SHLP2 enhanced mtGFP fluorescence intensity in amyloid-β_1–42_-treated (amyloid-β_1–42_ Active) AMD (7A bottom panel-5^th^ image; 451.02% increase-7C) and in amyloid-β_42–1_ SC-treated AMD (7A bottom panel-6^th^ image; 83.81% increase-7C). Data are presented as Mean ± SEM and normalized to untreated normal cybrids which were assigned a value of 1. Experiments were performed at the 72 hr time-point.

To investigate if in addition to preventing amyloid-β-induced cell death, SHLP2 also blocks amyloid-β-induced mitochondrial damage, the following experiments were conducted. The SHLP2- and amyloid-β-treated normal and AMD cybrids’ mitochondria were labeled with a GFP stain followed by cell imaging (Fig. 7A). Addition of amyloid-β_1–42_ (active form) led to a drastic decline in mtGFP fluorescence in normal (*77%*; *NL UN: 1* ± *0*.*15 a*.*u*. *vs*. *NL amyloid-β*_*1*–*42*_*: 0*.*23* ± *0*.*037 a*.*u*.; *P* < *0*.*001*, *n* = *5*) (Fig. 7B–bar 1 vs. bar 2) and AMD (*65%*; *AMD UN: 0*.*28* ± *0*.*035 a*.*u*. *vs*. *AMD amyloid-β*_*1*–*42*_*: 0*.*098* ± *0*.*0037 a*.*u*.; *P* < *0*.*05*, *n* = *5*)*cybrids* (Fig. 7C–bar 1 vs. bar 2). Furthermore, SHLP2 treatment resulted in significantly higher mtGFP fluorescence intensity in amyloid-β_1–42_-treated normal cybrids (*223*.*2%*; *NL amyloid-β*_*1*–*42*_*: 0*.*237* ± *0*.*037 a*.*u*. *vs*. *NL SHLP2* + *amyloid-β*_*1*–*42*_*: 0*.*766* ± *0*.*096 a*.*u*.; *P* < *0*.*01*, *n* = *5*) (Fig. 7B–bar 2 vs. bar 5) and AMD cybrids (*451.02% increase*; *AMD amyloid-β*_*1*–*42*_*: 0*.*098* ± *0*.*0037 a*.*u*. *vs*. *AMD SHLP2* + *amyloid-β*_*1*–*42*_*: 0*.*54* ± *0*.*06 a*.*u*.; *P* < *0*.*001*, *n* = *4–5*) (Fig. 7C–bar 2 vs. bar 5) than the ones treated with amyloid-β_1–42_ alone. No significant change in mtGFP fluorescence was found between untreated and amyloid-β_42–1_ SC (scrambled control)-treated normal cybrids (*NL UN: 1* ± *0*.*15 a*.*u*. *vs*. *NL amyloid-β*_*42*–*1*_ *SC: 1*.*057* ± *0*.*0559 a*.*u*.; *P* > *0*.*05*, *n* = *5*) (Fig. 7B–bar 1 vs. bar 3), or AMD cybrids (*AMD UN: 0*.*28* ± *0*.*035 a*.*u*. *vs*. *AMD amyloid-β*_*42*–*1*_ *SC: 0*.*315* ± *0*.*039 a*.*u*.; *P* > *0*.*05*, *n* = *4–5*) (Fig. 7C–bar 1 vs. bar 3). However, SHLP2 + amyloid-β_42–1_ SC–treated AMD cybrids showed significant increase in mtGFP staining intensity compared to the AMD cybrids treated with amyloid-β_42–1_ SC alone (83.81%; *AMD amyloid-β*_*42*–*1*_ *SC: 0*.*315* ± *0*.*039 a*.*u*. *vs*. *AMD SHLP2* + *amyloid-β*_*42*–*1*_ *SC: 0*.*579* ± *0*.*074 a*.*u*.; *P* < *0*.*01*, *n* = *4-5*) (Fig. 7C–bar 3 vs. bar 6). SHLP2 did not cause any significant change in mtGFP fluorescence between amyloid-β_42–1_ SC–treated normal cybrids (*NL amyloid-β*_*42*–*1*_ *SC: 1*.*057* ± *0*.*0559 a*.*u*. *vs*. *NL SHLP2* + *amyloid-β*_*42*–*1*_ *SC: 1*.*045* ± *0*.*068 a*.*u*.; *P* > *0*.*05*, *n* = *5*) (Fig. 7B–bar 3 vs. bar 6).

## Discussion

Identification of novel curative molecules that can mitigate retinal atrophy is critical to the development of a treatment regime for macular degeneration. Herein, we demonstrate that SHLP2, a naturally occurring MDP, plays a central role in protecting AMD retinal cells and mitochondria in an *in vitro* transmitochondrial ARPE-19 cell model.

The cytoprotective action of MDPs has been very well-characterized in various disease models including neurological diseases e.g., Alzheimer’s disease^10^, and retinal diseases e.g., macular degeneration^11^; and this has added new dimensions to the field of degenerative disease therapy. Humanin, the first characterized MDP, and its analogs have been shown to have therapeutic potential in *in vitro* and *in vivo* models of numerous diseases such as Alzheimer’s disease, amyotrophic lateral sclerosis, Parkinson’s disease, stroke, diabetes, cancer, cardiovascular diseases, and atherosclerosis^5,8,12–17^. In the current study, we found that the mtDNA-encoded *MT-RNR2* gene, which harbors ORFs for cytoprotective MDPs including Humanin and SHLPs, was down-regulated by 56% in AMD cybrids compared to normal cybrids. Other studies have reported that RPE cells from AMD patients have fragmented and damaged mtDNA^18,19^, which one can speculate lead to lower expression of *MT-RNR2* gene. Consistent with our findings, previous studies have shown that mitochondrial dysfunction due to hepatotoxicity can also lead to reduced *MT-RNR2* gene expression^20^. Furthermore, plasma levels of MDPs such as Humanin, SHLP2, and MOTS-c decline significantly with age, suggesting a correlation between loss of MDPs and the deteriorating biological processes associated with aging and diseases^8^.

The mechanisms by which MDPs are regulated are unclear. Interestingly, in comparison to untreated cybrids, exogenous addition of SHLP2 decreased *MT-RNR2* gene expression by 42.3% (P = 0.04) in AMD cybrids. This could support the idea that production of SHLP2 MDP may be regulated via a negative feedback loop, wherein the cellular system detects the exogenously added SHLP2 peptide and prevents the *MT-RNR2* gene from being expressed to maintain a healthy balance of MDPs within the cell. Although further studies are required to verify this feedback loop, to our knowledge, this is the first report to demonstrate the involvement of such a counterbalancing effect in MDP regulation.

Recently, Hinton *et al*. demonstrated that Humanin enhanced mitochondrial respiration via an increase in ATP levels, reserve capacity, oxygen consumption rate, and proton leak in RPE cells^21^. SHLP2 also improves mitochondrial metabolism by significantly increasing cellular ATP production and mitochondrial oxygen consumption rate^8^. In the present study, we compared the protein levels of OXPHOS Complex I-V subunits between untreated and SHLP2-treated AMD and normal ARPE-19 cybrid cells. The AMD cybrids showed drastically diminished protein levels of complex I-V subunits, suggesting compromised mitochondrial bioenergetics in the untreated AMD group compared to untreated normal. However, AMD cybrids treated with SHLP2 showed substantially augmented OXPHOS complex protein subunit levels as represented by an increase in the levels of NADH-coenzyme Q oxidoreductase (Complex I subunit) by 24%, Succinate-coenzyme Q oxidoreductase (Complex II subunit) by 59%, Coenzyme Q-cytochrome c oxidoreductase (Complex III subunit) by 37%, Cytochrome c oxidase (Complex IV subunit) by 46%, and ATP synthase (Complex V subunit) by 38%. Since these respiratory chain complexes play a vital role in ATP generation and maintenance of mitochondrial membrane potential, these findings indicate that SHLP2 preserves OXPHOS complex I-V protein subunits, thereby stabilizing AMD mitochondria. Interestingly, addition of SHLP2 did not show any considerable effect on electron transport chain complex I-V protein subunits’ levels in normal cybrids.

Prior MDP studies have shown that Humanin G increases mitochondria-targeted fluorescence staining intensity in AMD^4^. To test if SHLP2 has similar effects, cells were labeled with a baculovirus fusion construct stain containing GFP + mitochondrial leader peptide, which specifically targets mitochondria and stains independently of function (i.e., membrane potential). Our results revealed that SHLP2 treatment led to a 153.02% increase in mtGFP fluorescence intensity in AMD cybrids compared to their untreated counterparts. Furthermore, SHLP2-treated AMD cybrids showed 40.3% higher relative mtDNA copy numbers and 307.87% higher *PGC-1α* gene expression compared to untreated AMD cybrids. To our knowledge, this is the first study that reports the ability of SHLP2 peptide to increase mtDNA copies and enhance the gene expression of *PGC-1α*, a master regulator of mitochondrial biogenesis. These findings suggest that SHLP2 plays a crucial role in protecting mitochondrial function and number in AMD cybrids. This is important and supports therapeutic approaches because protecting mitochondrial structure and function is the key to maintaining cellular health in AMD.

One mechanism by which SHLP2 blocks cell death is through its inhibitory effects on apoptosis. AMD cybrids treated with SHLP2 showed a 21.79% increase in viable cell numbers compared to untreated AMD cybrids. The apoptosis pathway in SHLP2-treated cybrids was evaluated and showed reduced apoptosis, represented by down-regulation of effector caspases i.e., (1) decreased gene expression of *Caspase-3* by 81.8% and of *Caspase-7* by 72.48%, and (2) reduction in Cleaved Caspase-3 protein levels by 56.45% in AMD cybrids. These findings are consistent with a study by Cohen *et al*. demonstrating that SHLP2 enhanced cell survival and improved cell function in a pancreatic carcinoma cell line^8^. Moreover, other MDPs such as Humanin and its variants reportedly reduce apoptosis and improve cell survival in RPE cells^4,21^.

We found that another property of SHLP2 is to act as a rescue factor against amyloid-β induced cytotoxicity. Deposition of amyloid-β, a constituent of drusen, has been associated with AMD progression in the retina^22^ and with Alzheimer’s disease in the brain^23,24^. Sub-RPE deposition of amyloid-β increases with age and is associated with early and advanced stages of AMD^25^. Amyloid-β deposits lead to local RPE toxicity and inflammation, thereby contributing to drusen biogenesis and AMD pathogenesis. It is known that ocular amyloidosis also accumulates amyloid fibrils in the vitreous and the cornea^26^. In the present study, cybrids were treated with a form of active amyloid-β_1–42_ peptide that is a key contributor to Alzheimer’s disease pathology^27^. SHLP2 pretreatment in AMD cybrids led to 40.98% higher cell viability compared to the AMD cybrids treated with amyloid-β_1–42_ peptide alone. As a control, the amyloid-β_42–1_ scrambled peptide did not show any appreciable effect on the viable cell count of cybrids. In addition to improving the health of cells exposed to amyloid-β_1–42_, SHLP2 also protected against loss of AMD mitochondria induced by amyloid-β insult. Increased mitochondria-targeting mtGFP staining intensity was observed in SHLP2 + Amy- β_1–42_-treated cybrids. Our observations are consistent with a study by Ding *et al*. wherein anti-amyloid therapy served as a viable option for protection against retinal and visual function damage, and reduced ocular amyloid-β deposits in a macular degeneration model^28^.

In summary, our study presents novel findings that delineate the role of SHLP2 as a mitochondria-targeting protective molecule that confers both morphological and functional protection against cellular and mitochondrial toxicity. These observations make SHLP2 a promising prospect as a therapeutic for macular degeneration. Further studies are required for a smooth transition of the SHLP2 peptide as a therapeutic option from lab bench to clinic.

## Methods

### Human subjects

Our research that involved human subjects was approved by the Institutional Review Board of the University of California Irvine (Approval #2003–3131). Clinical investigations were performed according to the tenets of Declaration of Helsinki and informed written consent was obtained from all participants.

### Cell culture

Normal and AMD ARPE-19 transmitochondrial cells used in this study were prepared by polyethylene glycol fusion of mitochondrial DNA-deficient APRE-19 (Rho0) cell line with platelets isolated from either AMD patients or age-matched normal subjects^29^, as described previously^4^. All ARPE-19 transmitochondrial cybrids belonged to the ‘H’ mitochondrial DNA haplogroup. Passage 5 cybrids were used for all experiments. Age-matched normal cybrids served as controls.

### Treatment with SHLP2

Stock solution of SHLP2 was prepared by reconstitution of lyophilized SHLP2 (Anaspec, Fremont, CA) in water. SHLP2 stock solution was further dissolved in culture media to obtain a working concentration of 3.2 µM. In this study, all cybrids were treated with 3.2 µM SHLP2.

### MTT assay

The colorimetric MTT assay that is based on reduction of tetrazolium salts was used to measure cell viability. This assay is based on the principle that actively metabolizing healthy cells contain NAD(P)H-dependent cellular oxidoreductases, which reduce the yellow tetrazolium MTT [3-(4,5-dimethylthiazol-2-yl)-2,5-diphenyltetrazolium bromide] dye to its insoluble purple-colored formazan. Dimethyl sulfoxide (DMSO) is then added to solubilize the formazan crystals and the colorimetric signal, which is proportional to the number of viable cells, is determined by measurement of optical density at 570 nm.

For this assay, cells were plated in 96-well plates and were treated with MTT solution (Cat. # 30006, Biotium, CA, USA) at 37 °C for 1 hr. Signal absorbance was measured on a spectrophotometer at 570 nm and background absorbance was measured at 630 nm. Background absorbance was subtracted from signal absorbance to obtain normalized absorbance values. Absorbance at 630 nm is background absorbance of the non-reduced MTT itself, before it is reduced into the formazan that absorbs at 570 nm. Therefore the 630 nm reading is a measurement of the amount of MTT in each well. The colorimetric signal obtained was proportional to the cell number.

### CellLight mtGFP staining and Confocal microscopy

The CellLight Mitochondrial GFP reagent (Cat. # C10600, Thermo Fisher Scientific, MA, USA) was used to estimate the mitochondrial number in normal and AMD cybrid cells. CellLight mtGFP is a baculovirus fusion construct having a mammalian promoter and a leader sequence of E1 alpha pyruvate dehydrogenase fused to GFP. It specifically targets mitochondria and mtGFP fluorescence can be quantified. Cells were plated in 4-well chamber slides, transduced with CellLight mtGFP reagent, and incubated overnight at 37 °C. Cells were then washed with 1X TBS (Tris buffered saline), fixed in paraformaldehyde, and mounted in DAPI. Confocal z-stack images were captured using the LSM-700 Confocal microscope (Zeiss, Thornwood, NY, USA). Images were quantified using ZEN 2 lite software (Zeiss). Each image was a maximum intensity projection. Each mtGFP image was normalized to DAPI (internal control).

### Mitochondrial DNA (mtDNA) copy number

Total DNA was isolated from normal and AMD cells and qRT-PCR was performed using 18S (nuclear) and MT-ND2 (mitochondrial) TaqMan gene expression assays (Cat. # 4331182, Thermo Fisher Scientific) and TaqMan gene expression master mix (Cat.# 4369016, Thermo Fisher Scientific). Relative mtDNA copy numbers were determined using delta Cts. All samples were run in triplicates.

### Quantitative Real-Time PCR (qRT-PCR)

RNA was extracted from cells using the RNeasy Mini Kit (Qiagen, Valencia, CA, USA) and quantified using NanoDrop 1000 Spectrophotometer (Thermoscientific, Waltham, Massachusetts, USA). 100 ng/µL of RNA was reverse transcribed into cDNA using Superscript VILO Master Mix (Cat. #11755–050, Invitrogen, Grand Island, NY, USA).

StepOnePlus Real-Time PCR system (Applied Biosystems, Carlsbad, CA, USA) was used to perform qRT-PCR. Power SYBR® Green PCR Master Mix (Cat. # 4367659, Life Technologies, Grand Island, NY, USA) and the QuantiTect Primer Assays were used to study the expression of *Caspase-3* gene (Cat. # QT00023947, Qiagen), and *Caspase-7* gene (Cat. # QT00003549, Qiagen). *PGC-1α* gene expression was examined using *PGC-1α* KiCqStart® SYBR® green primer (Cat. # kspq12012, Sigma, St. Louis, MO). Specific housekeeper genes used were *HPRT1* (Cat. # QT00059066, Qiagen), *ALAS variant 1* (Cat. # QT01160467, Qiagen), and *HMBS* (Cat. # QT00014462). TaqMan gene expression master mix (Cat. # 4369016, Life Technologies) and TaqMan gene expression assays were used to examine the expression of the *MT-RNR2* gene (Assay ID: Hs02596860_s1, Life Technologies), for which *GAPDH* (Assay ID: Hs02786624_g1, Life Technologies) (Table S1) was used as a housekeeper gene. Data analysis was performed using ∆∆Ct method which was calculated by subtracting ∆Ct of the AMD group from ∆Ct of the normal group. ∆Ct was the difference between the Cts (threshold cycles) of the target gene and Cts of the housekeeper gene (reference gene). Fold change was calculated using the following formula: Fold change = 2^ΔΔCt^.

### Western blotting analyses

Cells were lysed using RIPA buffer (Cat. # 89900, Life Technologies), supernatant was transferred to a new microfuge tube, and Bio-Rad Dc protein assay kit (Bio-Rad Laboratories, Richmond, CA) was used to measure protein concentration. Total protein samples were loaded in equal concentrations into the wells of 4–12% Bolt mini gels (Life Technologies) followed by SDS-PAGE electrophoresis. Gels were then transferred onto PVDF membranes, blocked in 5% milk for 1 hr, then incubated overnight at 4°C in the following primary antibodies: Total OXPHOS Human WB Antibody Cocktail (Cat. # ab110411, Abcam, Cambridge, MA, USA) and Cleaved Caspase-3 Ab (Cat. # 9661 T, Cell Signaling Technology (CST), Danvers, MA, USA). Washing the blots with 1X TBST (Tris Buffered Saline-Tween20) was followed by incubation with the respective secondary antibodies: Anti-mouse IgG (HRP) Ab (Cat. # 7076, CST) for OXPHOS, and anti-rabbit IgG (HRP) Ab (Cat. # GTX 213110-01, Genetex, Irvine, CA, USA) for Cleaved Caspase-3. Primary and secondary antibodies were diluted in 1X TBST. Following secondary antibody incubation, the blots were washed with 1X TBST. Clarity™ Western ECL Blotting Substrate (Cat. # 1705060, Bio-Rad) was used for development of blots and Versadoc imager (Bio-Rad) was used to detect protein bands. β-actin antibody (Cat. # GTX 110564, Genetex) was used as a loading control for all Western blotting experiments, and all data were normalized to β-actin before being normalized to NL untreated samples. Densitometry measurements were performed using Image J software (NIH Image).

### Treatment with Amyloid-β peptides

Stock solutions of amyloid-β_1–42_ (active form) (Cat.# AS-20276, Anaspec) and amyloid-β_42–1_ (inactive scrambled control) peptides (Cat.# AS-27276, Anaspec) were prepared by reconstituting the lyophilized powder in 1% ammonium hydroxide. This stock was subsequently dissolved in 1X PBS (Phosphate Buffered Saline) to obtain a 20 µM working solution. Cells were treated with 20 µM amyloid-β peptides for 24 hr.

### Statistical analysis

Unpaired Student’s t-test (2 groups) or one-way ANOVA (3 or more groups) followed by post-hoc Tukey–Kramer test (GraphPad Prism 5.0; GraphPad Software, CA, USA) were used to analyze data between groups. P values < 0.05 were considered statistically significant.

## Electronic supplementary material

Supplementary Information
