# Supplementary material for: Characterizing the protective effects of SHLP2, a mitochondrial-derived peptide, in macular degeneration
Source: Sci Rep. 2018 Oct 11;8:15175. doi: 10.1038/s41598-018-33290-5 (PMC6182005; doi:10.1038/s41598-018-33290-5)
Supplement: Supplementary file 1 — Supplementary Information [file 41598_2018_33290_MOESM1_ESM.pdf]

Characterizing the protective effects of SHLP2, a mitochondrial-derived peptide, in macular degeneration

Sonali Nashine[1], Pinchas Cohen[2], Anthony B. Nesburn[1,3], Baruch D. Kuppermann[1], M. Cristina Kenney[1,4*]

1. Department of Ophthalmology, Gavin Herbert Eye Institute, University of California Irvine, Irvine, CA, USA.
2. Leonard Davis School of Gerontology, University of Southern California, Los Angeles, CA, USA.
3. Cedars-Sinai Medical Center, Los Angeles, CA, USA.
4. Department of Pathology and Laboratory Medicine, University of California Irvine, Irvine, CA, USA.

[*]**Corresponding Author:** M. Cristina Kenney, M.D., Ph.D.
Gavin Herbert Eye Institute, Ophthalmology Research Laboratory
University of California Irvine, Hewitt Hall, Room 2028
843 Health Science Road
Irvine, CA 92697
Telephone (949) 824-7603
Fax (949) 824-9626
Email mkenney@uci.edu

Supplementary Information

Figure S1 A

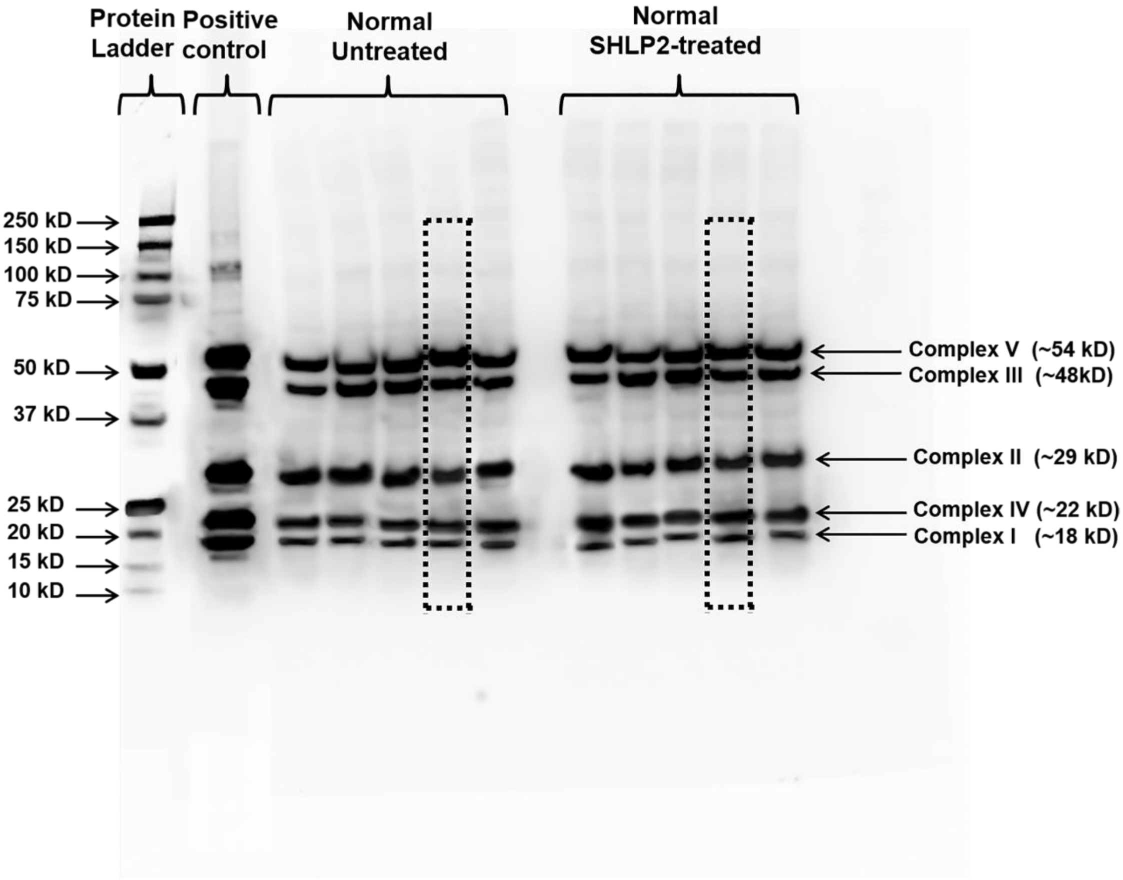

Figure S1 A legend: Full-length Western blot showing OXPHOS complex subunit proteins in normal (NL) untreated and normal (NL) SHLP2-treated samples. The dotted lines demarcate the lanes that were used as representative images in the main text. Positive control is human heart mitochondria Western blot control that was provided with the antibody cocktail.

Figure S1 B

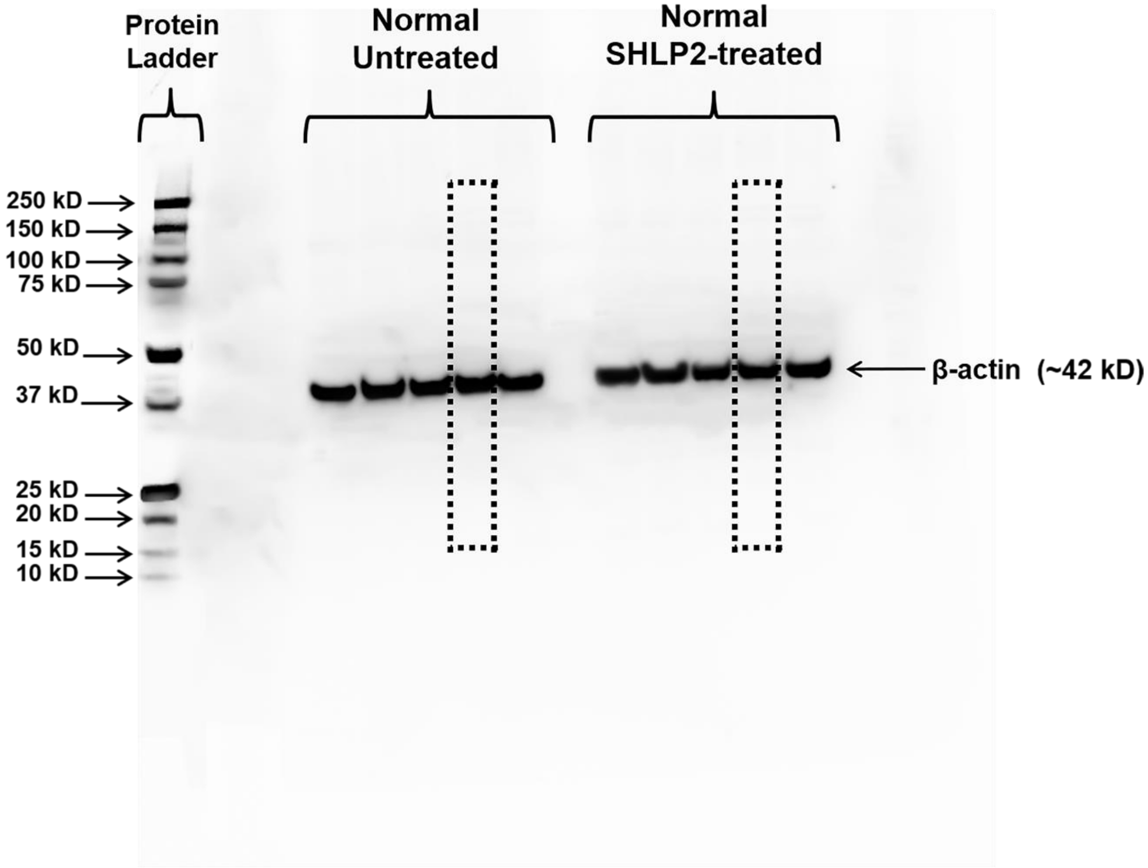

Figure S1 B legend: Full-length Western blot showing β-actin (loading control) bands for OXPHOS complex subunit proteins in normal untreated and normal SHLP2-treated samples. Loading control β-actin was run on the same gel. The dotted lines demarcate the lanes that were used as representative images in the main text.

Figure S2 A

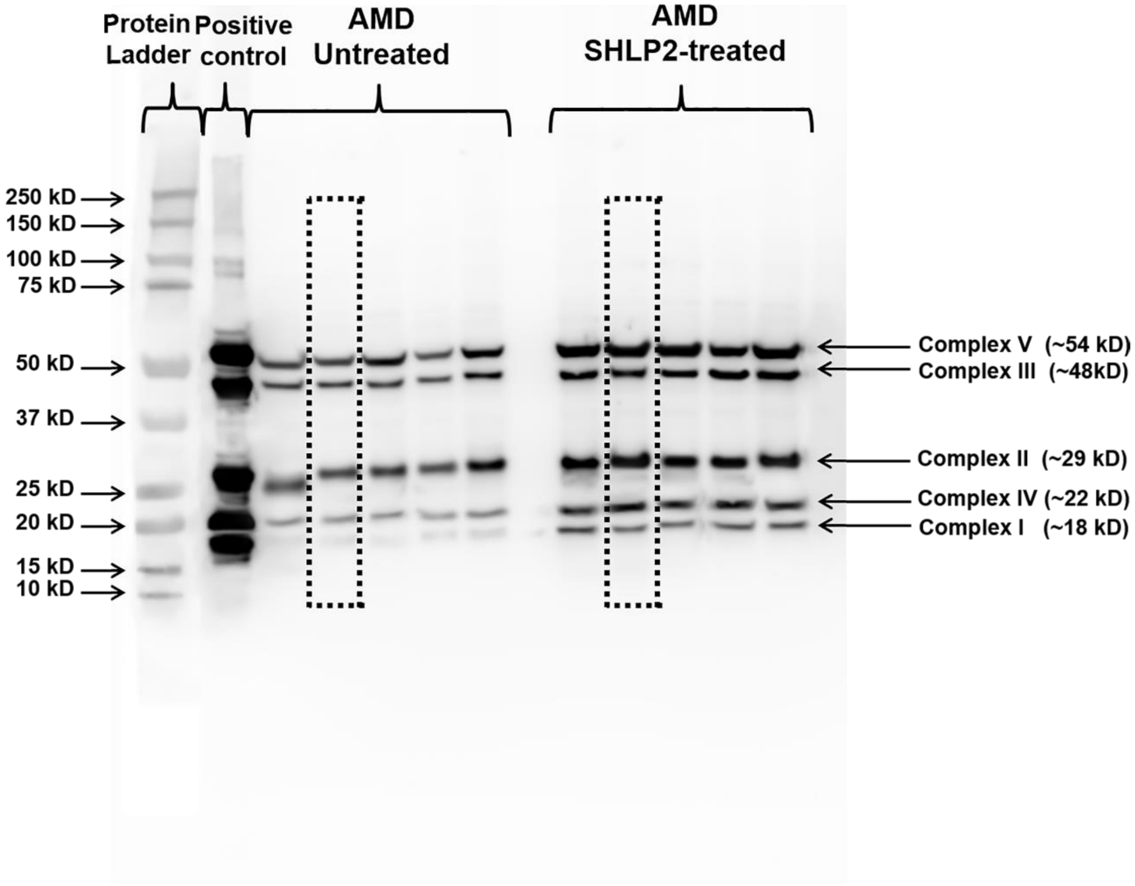

Figure S2 A legend: Full-length Western blot showing OXPHOS complex subunit proteins in AMD untreated and AMD SHLP2-treated samples. The dotted lines demarcate the lanes that were used as representative images in the main text. Positive control is human heart mitochondria Western blot control that was provided with the antibody cocktail.

Figure S2 B

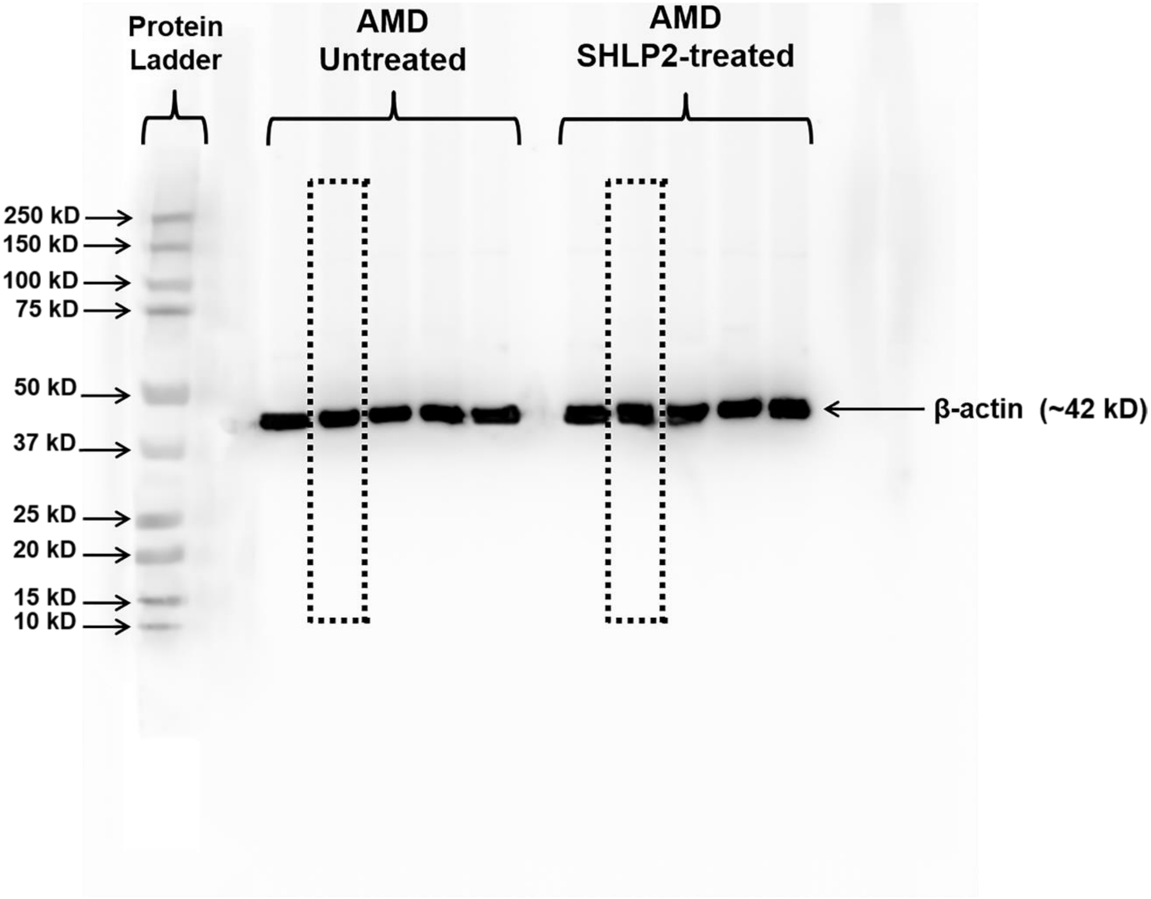

Figure S2 B legend: Full-length Western blot showing β-actin (loading control) bands for OXPHOS subunit proteins in AMD untreated and AMD SHLP2-treated samples. Loading control β-actin was run on the same gel. The dotted lines demarcate the lanes that were used as representative images in the main text.

Figure S3 A

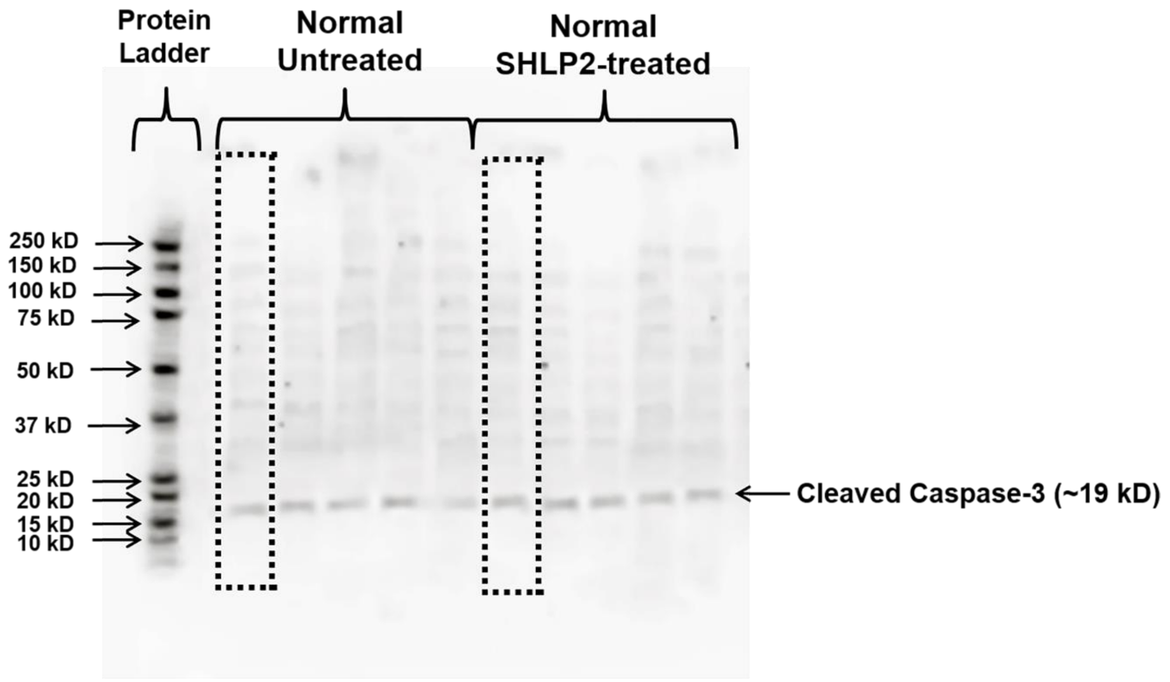

Figure S3 A legend: Full-length Western blot showing cleaved Caspase-3 protein bands in normal untreated and normal SHLP2-treated samples. The dotted lines demarcate the lanes that were used as representative images in the main text.

Figure S3 B

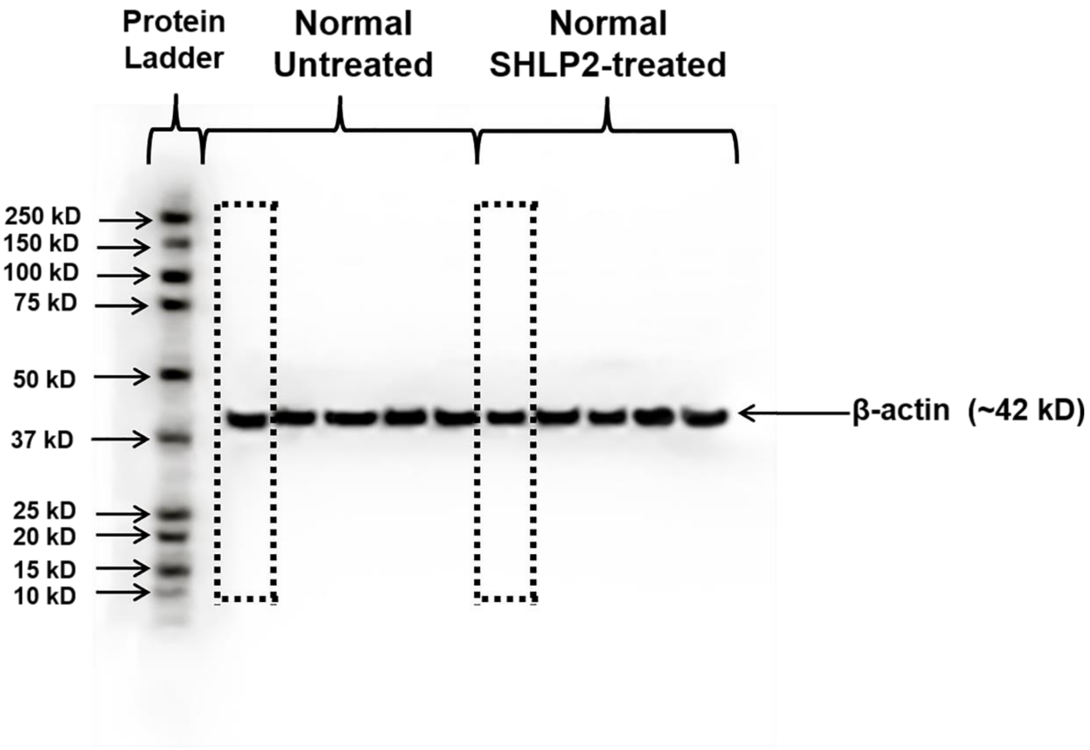

Figure S3 B legend: Full-length Western blot showing β-actin for cleaved Caspase-3 protein bands in normal untreated and normal SHLP2-treated samples. The dotted lines demarcate the lanes that were used as representative images in the main text.

Figure S4 A

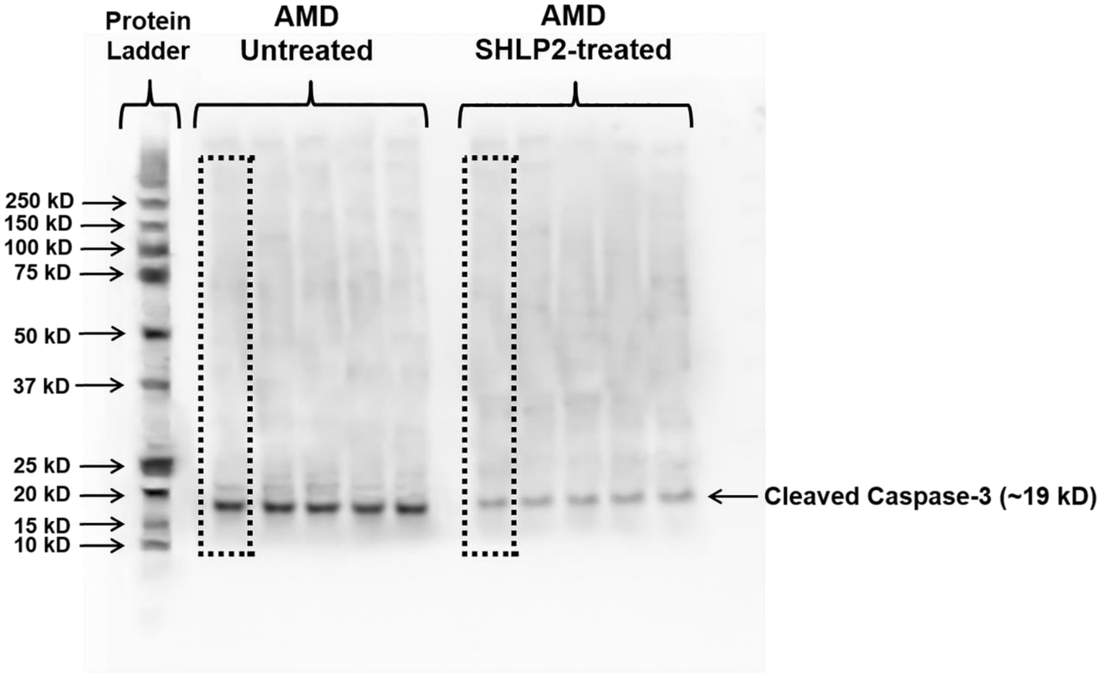

Figure S4 A legend: Full-length Western blot showing cleaved Caspase-3 protein bands in AMD untreated and AMD SHLP2-treated samples. The dotted lines demarcate the lanes that were used as representative images in the main text.

Figure S4 B

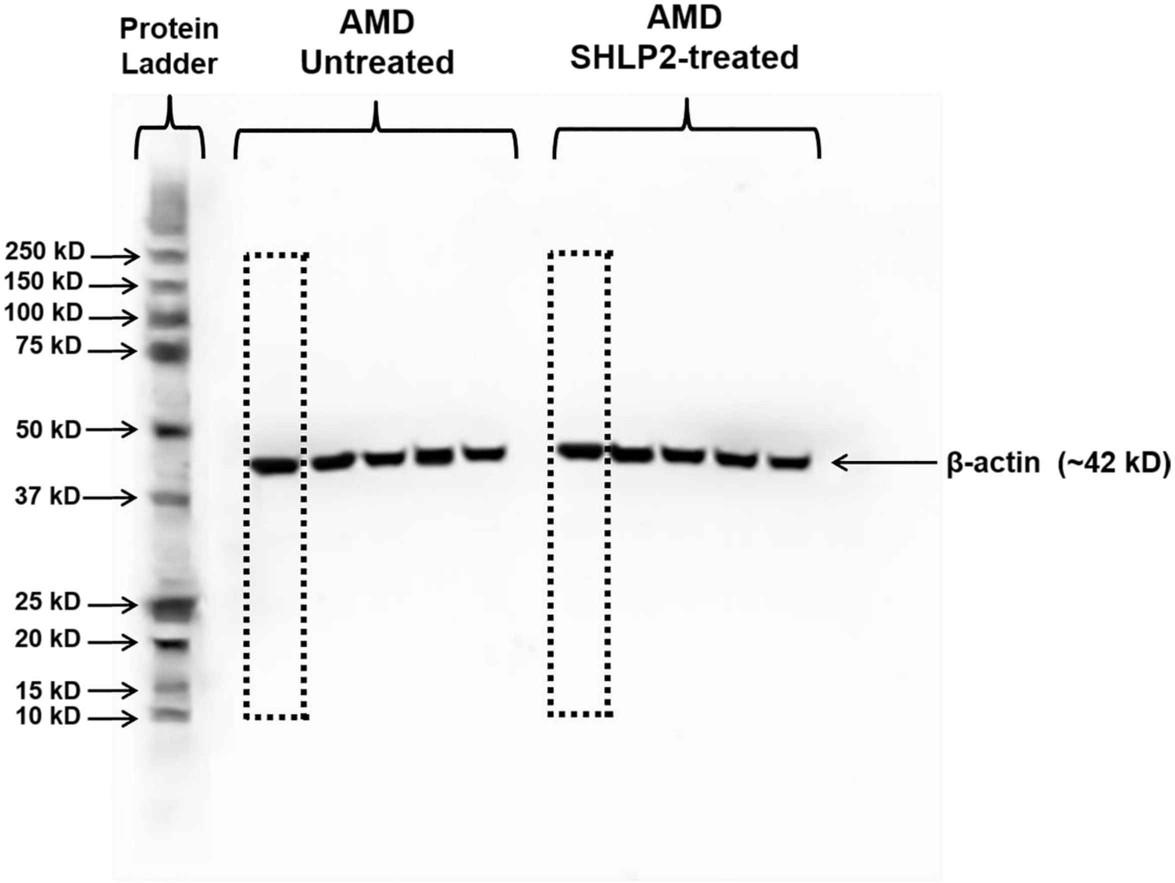

Figure S4 B legend: Full-length Western blot showing β-actin (loading control) bands for cleaved Caspase-3 protein in AMD untreated and AMD SHLP2-treated samples. Loading control β-actin was run on the same gel. The dotted lines demarcate the lanes that were used as representative images in the main text.

Table S1.

Primer name	Range of Cycle threshold	Housekeeping gene	Range of Cycle threshold
Caspase-3	**20-22**	*HPRT1*	**20-22**
Caspase-7	**25-29**	*ALAS variant 1*	**25-28**
PGC-1α	**21-28**	*HMBS*	**21-28**
MT-RNR2	**14-15**	*GAPDH*	**18-19**

Table S1 legend: Table showing names and cycle threshold (Ct) range of all qRT-PCR primers used for gene expression studies.
